# Enhancing Anti-Tumor Efficacy of Doxorubicin by Non-Covalent Conjugation to Gold Nanoparticles – *In Vitro* Studies on Feline Fibrosarcoma Cell Lines

**DOI:** 10.1371/journal.pone.0124955

**Published:** 2015-04-30

**Authors:** Michał Wójcik, Wiktor Lewandowski, Magdalena Król, Karol Pawłowski, Józef Mieczkowski, Roman Lechowski, Katarzyna Zabielska

**Affiliations:** 1 Faculty of Chemistry, University of Warsaw, Warsaw, Poland; 2 Faculty of Veterinary Medicine, Warsaw University of Life Sciences, Warsaw, Poland; Brandeis University, UNITED STATES

## Abstract

**Background:**

Feline injection-site sarcomas are malignant skin tumors of mesenchymal origin, the treatment of which is a challenge for veterinary practitioners. Methods of treatment include radical surgery, radiotherapy and chemotherapy. The most commonly used cytostatic drugs are cyclophosphamide, doxorubicin and vincristine. However, the use of cytostatics as adjunctive treatment is limited due to their adverse side-effects, low biodistribution after intravenous administration and multidrug resistance. Colloid gold nanoparticles are promising drug delivery systems to overcome multidrug resistance, which is a main cause of ineffective chemotherapy treatment. The use of colloid gold nanoparticles as building blocks for drug delivery systems is preferred due to ease of surface functionalization with various molecules, chemical stability and their low toxicity.

**Methods:**

Stability and structure of the glutathione-stabilized gold nanoparticles non-covalently modified with doxorubicin (Au-GSH-Dox) was confirmed using XPS, TEM, FT-IR, SAXRD and SAXS analyses. MTT assay, Annexin V and Propidium Iodide Apoptosis assay and Rhodamine 123 and Verapamil assay were performed on 4 feline fibrosarcoma cell lines (FFS1WAW, FFS1, FFS3, FFS5). Statistical analyses were performed using Graph Pad Prism 5.0 (USA).

**Results:**

A novel approach, glutathione-stabilized gold nanoparticles (4.3 +/- 1.1 nm in diameter) non-covalently modified with doxorubicin (Au-GSH-Dox) was designed and synthesized. A higher cytotoxic effect (p<0.01) of Au-GSH-Dox than that of free doxorubicin has been observed in 3 (FFS1, FFS3, FFS1WAW) out of 4 feline fibrosarcoma cell lines. The effect has been correlated to the activity of glycoprotein P (main efflux pump responsible for multidrug resistance).

**Conclusions:**

The results indicate that Au-GSH-Dox may be a potent new therapeutic agent to increase the efficacy of the drug by overcoming the resistance to doxorubicin in feline fibrosarcoma cell lines. Moreover, as doxorubicin is non-covalently attached to glutathione coated nanoparticles the synthesized system is potentially suitable to a wealth of different drug molecules.

## Introduction

The unique properties of nanomaterials make them an important technological target of research for pharmaceutical industry.[1] Exact diagnostic imaging, precise targeting, and improved performance of therapeutic agents can all be achieved using systems based on nanostructures.[2] Drug vectorization by nanomaterials is especially important in the context of growing multidrug resistance (MDR) of tumors, which is a significant cause of ineffective chemotherapy treatment.[3] Among various mechanisms of MDR, such as increased metabolism of the therapeutic, lowering the drug uptake or enhanced repair DNA damage,[4] the major factor responsible for MDR is high activity of efflux pumps (especially P glycoprotein (P-gp), multidrug resistance protein 1 (MDRP1)). Recently, the use of nanomaterials as drug delivery systems (DDS) was indicated to be a solution of the MDR problem, increasing the potential of available chemotherapeutics.[5–9] However, these systems usually have complex, multilayer structure which increases production costs and decreases applicative potential. Therefore, in our studies, we have aimed at synthesizing delivery system capable of avoiding multidrug resistance, which would be characterized by structural simplicity.

In the process of design of DDS three issues should be addressed—nanoparticles (NPs) material, the drug molecule and the way to bind those two. Various materials were proposed as delivery platforms: metallic and magnetic nanoparticles, cyclodextrin and liposome-based materials, polymeric vesicles, carbon-based structures and dendrimers. [10–16] The use of colloid gold nanoparticles as building blocks for DDSs is preferred due to the ease of surface functionalization with various molecules, chemical stability and their low toxicity.[17] Although few examples indicating toxicity of Au NPs were reported up-to-date, the effect can be attributed to the toxicity of free ligands released from nanoparticle surface [18] and limited to very small particles.[19] To tackle this issue we propose to use a biogenic molecule, glutathione, as the stabilizing agent. We were encouraged by recent research evidencing low immunogenicity, clearance times, and biocompatibility of glutathione-coated Au nanoparticles.[20] Also, we propose to focus on nanoparticles with diameter above 2 nm.

Versatility of Au nanospheres as a DDS platform is further supported by examples of various drug vectorization—paclitaxel,[21] ciprofloxacin,[22] doxorubicin (Dox),[7,9,23–26] curcumin,[27] and chloroquine.[28] Importantly, in some of these research authors highlighted the ability of the systems to efficiently avoid P-gp, evidencing the potential of Au NPs to overcome MDR.[7,9]

Feline injection-site sarcomas (ISS) are the second most common malignant skin tumors in cats. In 20% of patients they give metastasis to lungs and their treatment is a challenge for veterinary practitioners. Although the first method of treatment is radical surgery—with at least 3 cm margin of safety tissue—not all tumors can be removed surgically, depending on the localization (intrascapular with infiltration on thorax part of spinal cord) and the size of the tumor and above all the condition of the patient. Adjunctive treatment includes radiotherapy and chemotherapy. It has been proven that chemotherapeutic agents such as doxorubicin, cyclophosphamide and vincristine show high efficiency against soft tissue sarcomas, including feline injection-site sarcomas. In our efforts to design DDS we have chosen to deliver doxorubicin as the drug molecule, since it is the first choice therapeutic agent in soft tissue sarcomas. However, Dox effectiveness is debatable,[1,29–31] it has many adverse side effects such as nephrotoxicity and mielosupression[32] due to its low biodistribution, while in water solutions it shows low stability and rapid uptake by macrophages of reticuloendoepithelial system after intravenous administration. Therefore, improving the action of Dox would be of great importance for ISS.

The last issue to be considered in DDS design is the strategy allowing drug attachment to the delivery platform. Covalent and non-covalent systems have been reported up-to-date.[33,34] In the former, most popular approach, payload delivery relies on stimuli responsive bonds, capitalizing e.g. on pH difference between cancer and healthy cells.[9,35,36] Unfortunately, such structures pose the necessity to modify the drug molecule and therefore are prone to all risks related to a prodrug use.[17] On the contrary, the use of non-covalent interactions in drug delivery, allows avoiding these issues as well as renders the nanoparticle system more versatile.[17] Various studies have shown that for skin tumors non-covalent nanoparticle systems may be effectively administered via intratumoral injection, thus, reduce negative side effects, which are so common after standard intravenous administration. Beyond the biocomplex design, the main objective of this study was to assess the anti-tumor effect of glutathione-stabilized gold nanoparticles non-covalently modified with doxorubicin for feline injection-site fibrosarcoma cell lines and investigate the correlation between the P-gp activity of tested cell lines and cytotoxic effect of the synthesized DDS.

## Materials and Methods

### 2.1 Synthesis of glutathione-stabilized gold nanoparticles (Au-GSH)

Gold chloride solution (93.0 μL, Sigma Aldrich) was added to a distilled water (26 mL) in a round-bottom flask; the solution was cooled to ca. 0°C in an ice bath and glutathione (GSH, 162 mg) was slowly added to the flask under vigorous stirring, resulting in a color change of the solution from clear yellow to cloudy white, indicating the formation of the auric-polymer. The mixture turned clear after continuous stirring and addition of 3.2 mL of saturated NaHCO_3_. The mixture was then cooled to 0°C in an ice bath for 10 min and aqueous solution of NaBH_4_ (0.26 M, 5.0 mL) was rapidly injected into the above mixture under vigorous stirring and allowed to react for another hour. Then, MeOH (32 mL) was slowly added under stirring to precipitate nanoparticles. The material was collected by centrifugation (5000 rpm, 10 min), washed with 1 mL MeOH/H_2_O (1:2, v/v), repeatedly washed by methanol (1 mL ×3) and dried under reduced pressure at room temperature. Fractions of smaller gold nanoclusters, were washed off by further precipitation/centrifugation protocol of the nanoparticles in methanol/water mixtures, as reported earlier.[37] The resultant material are gold nanoparticles with glutathione molecules covalently attached to the surface via mercapto-moiety.[17, 37] Based on x-ray photoelectron spectroscopy measurements (as shown below) we can estimate that ca. 16% of the mass of the as obtained material can be attributed to organic coating layer. This is in agreement with theoretical calculations [38] which indicate that single nanoparticle having ca. 4.4 nm diameter is composed 2951 Au atoms and 371 surface ligands, which in our case can be translated to 584 and 114 kDa mass, respectively. Since mean mass of the obtained Au-GSH material was ca. 26.6 mg then 22.3 mg can be attributed to Au. Since 25.9 mg of Au was used for the synthesis (in the form of gold chloride solution) therefore yield of the reaction relative to gold is ca. 87%.

### 2.2 Non-covalent conjugation of GSH-stabilized nanoparticles with doxorubicin (Au-GSH-Dox)

Au-GSH solid (100 mg) was dissolved in 10 mL of distilled water and then 6 mg of Doxorubicin hydrochloride (Sigma Aldrich) was added. The mixture was stirred for 2 days under inert atmosphere at room temperature. The final pH of the reaction mixture was close to 7. Purification of the resultant Au-GSH-Dox was performed by centrifugation at 4000 rpm for 50 min using 10 kDa Amicon Ultra centrifuge filter. The retentate was rinsed with phosphate buffer (0.1 M, pH = 7). Rinsing was repeated until the concentration of free Doxorubicin hydrochloride in permeate was not detectable via UV-Vis. The product was finally redispersed in distilled water. For biological experiments nanoparticles were added to standard PBS buffer solutions with pH ca. 7.4. Nanoparticles were characterized with x-ray photoelectron spectroscopy (XPS) and transmission electron microscopy (TEM).

### 2.3 Analysis of nanoparticles

#### 2.3.1 XPS analysis of nanoparticles

Nanoparticle samples were analyzed using a ESCALAB-210 X-ray photoelectron spectrometer (VG Scientific, UK). The size of the analyzed area was about 4 mm^2^. Non-monochromatized Al Ka = 1486.6 eV radiation was used for excitation (the lamp parameters 14.5 kV, 20 mA) under lowered pressure (5*10^–9^ mbar). Survey spectra was recorded for energies 1350 eV to 0 eV with 0.4 eV intervals. CasaXPS software was used for results interpretation. XPS analyses of Au-GSH and Au-GSH-Dox were collected under similar conditions.

#### 2.3.2 FT-IR analysis of nanoparticles

Nanoparticle samples were analyzed using a Nicolet 6700FT-IR spectrometer. Nanoparticle solution was drop-casted KBr pellets.

#### 2.3.3 TEM analysis of nanoparticles

Transmission electron microscopy (TEM) was performed using Zeiss Libra 120 instrument, with LaB6 cathode, equipped with OMEGA internal columnar filters and CCD camera. Solutions of nanoparticles were drop-casted onto holey carbon TEM grids and dried with argon.

#### 2.3.4 SAXRD and SAXS analysis of nanoparticles

The small angle X-ray diffraction (SAXRD) patterns for the powder samples were obtained with the BrukerNanostar system. The CuKα radiation was used, patterns were registered with an area detector VANTEC2000. The temperature of the sample was controlled with precision of 0.1 K. Kapton tape was used as a substrate for nanoparticle measurements. For measurements a solution of gold nanoparticles was drop-casted onto Kapton tape and dried with argon. The same Bruker Nanostar system was also used for the scattering experiments (SAXS). The scattering data from nanoparticle solution in water were analyzed using NANOFIT software, assuming spherical form factor for non-interacting gold particles (structure factor S = 1) and Shultz distribution of the particle sizes.

### 2.4 Cell lines

Four feline fibrosarcoma cell lines were used in this study (FFS1WAW, FFS1, FFS3 and FF35). FFS1WAW were derived in the Warsaw University of Life Sciences according to the protocol described in our previous studies.[39] FFS1, FFS3 and FFS5 cell lines were derived by Erlichsen et al.[40] Cells were cultured in standard conditions (in an atmosphere of 5% CO_2_, 95% humidified air at 37°C) in DMEM-high glucose medium (Gibco BRL, Scotland) enriched with 10% heat-inactivated fetal bovine serum (FBS, Sigma Aldrich, USA) and antibiotics: Penicillin with Streptomycin (50 I.U./mL, Gibco BRL, Scotland), Amphotericin B (2.5 mg/mL, Gibco BRL, Scotland) and grown in tissue culture flasks (Becton Dickinson, USA). The medium was changed every 48–72 hours (depending on confluence of cells) in order to assure optimal conditions for cell growth. When cell confluence reached 80–90%, as observed using phase contrast microscope (Olympus IX 70, Olympus Optical Co.), cells were passaged.

### 2.5 MTT assay

MTT assay was performed to assess the cytotoxic effect of Dox, Au-GSH-Dox and Au-GSH NPs.

Cells in the log phase of growth were trypsinized and seeded into 96-well plates (Nunc Inc., Denmark) at the concentration of 6 x 10^6^. When the confluence reached 70–80% medium was replaced with the medium containing examined compounds at nine various concentrations: 1; 10; 100; 250; 500; 1000; 2500; 5000; 10000 ng/ml for free Dox and the bound Dox in Au-GSH-Dox and 20; 200; 2000; 5000; 10000; 20000; 50000; 100000; 200000 ng/ml for Au. The doses of Au-GSH-Dox were 20 times higher than doses of Dox since Dox:Au ratio in Au-GSH-Dox was 1:20. No substances were added to the medium of a control group. After 24 hours of treatment with examined compounds cells were incubated for 4 hours in 0.5 mg/mL tetrazolium salt (MTT) diluted in phenol-red free RPMI medium (Sigma Aldrich, USA). 100 μl of DMSO was added to each well in order to complete solubilisation of the formazan crystals. Cells viability was quantified by measuring photometric absorbance at 570 nm using multi-well plate reader Infinite 200 PRO Tecan (TECAN, Mannedorf, Switzerland). For each concentration 3 technical measures were performed, experiment was conducted 3 times (n = 9).

### 2.6 Annexin V and Propidium Iodide Apoptosis Assay

Annexin V-FITC and Propidium Iodide (PI) dual staining was performed to assess the apoptosis in fibrosarcoma cells induced by treatment with examined compounds. FFS1WAW, FFS1 and FFS3 cells were incubated in 6-well plates until they reached 70–80% of confluence, then the medium was replaced with the medium containing examined compounds at the concentrations determined as IC50 with the MTT assay (Table 1). Control cells were incubated in a normal medium. After 24 hours of incubation cells were harvested by trypsinization and together with the cells floated in medium were stained using an Annexin V Kit (Becton Dickinson, USA), according to the manufacturer’s protocol. Then the cells were analysed by flow cytometry (BD FACS Aria II (Becton Dickinson, USA) within an hour after staining using a blue laser (488 nm) with three detection filters 480/10 (SSC/FSC), 530/30 (FITC) and 610/20 (PI). Early apoptotic cells with exposed phosphatidylserine but intact cell membranes bound only to Annexin V-FITC. Cells in late apoptotic stages were labeled with both Annexin V-FITC and PI, whereas necrotic cells were labelled with PI only.[41] All samples were assayed in triplicate, and each experiment was performed three times (n = 9).

**Table 1:** Activity of efflux pumps and half maximal inhibitory concentration (IC50) of Dox and DoxAu for feline fibrosarcoma cell lines (FFS1WAW, FFS1, FFS3, FFS5).

cell line	IC50 Dox (μg/ml)	IC50 Au-GSH-Dox* (μg/ml)	Activity of efflux pumps (after inhibition with 10 μM and 20 μM verapamil)
**FFS1WAW**	69.66	30.617 (p<0.05)	53
**FFS1**	35.39	2.815 (p<0.001)	385
**FFS3**	18.09	2.567 (p<0.001)	541
**FFS5**	8.67	10.218 (p = 0)	0

*IC50Au-GSH-Dox = IC50 of bound Dox in the Au-GSH-Dox

p values—compared IC50 of bound Dox in the Au-GSH-Dox to IC50 of free Dox

### 2.7 Test with rhodamine 123 and verapamil

The test was performed to assess the activity of efflux pumps (especially P-gp) which are responsible for MDR.[41,42] Cells with high efflux pumps activity exhibit low rhodamine 123 accumulations due to its active transport outside the cells. In order to assess the activity of efflux pumps, its blocker verapamil was added to the culture and the ratio of rhodamine 123 inside the cells after and before was examined. Higher ratio indicate higher activity of efflux pumps and thus, higher multidrug resistance of the cells.[43] Cells were incubated for an hour in 37°C with a presence of verapamil (10 μM either 20 μM) and 1uM of rhodamine 123. Control cells were incubated in 1uM of rhodamine 123. Then, cells were washed twice and resuspended in cold PBS and kept in 4°C in the dark until flow cytometry was performed. Results were analyzed using flow cytometer (BD FACS Aria II, Becton Dickinson). Cells shown in forward scatter and side scatter were gated and acquired through the fluorescence channel. The amount of fluorescence was plotted as a histogram within the gate. Data acquisition was performed using BD FACS Diva software (Becton Dickinson, USA) to determine mean fluorescence intensity values. The blue laser (488 nm) with detection filters 480/10 (SSC/FSC) and 530/30 for rhodamine 123 was used. The efflux pump activity was measured according to the formula [43]:

$$\left(MFI_{FFS\;rhodamine}{}_{+verampil}+MFI_{FFS\;rhodamine}\right)/MFI_{FFS\;rhodamine}$$

MFI- median fluorescence intensity

### 2.8 Statistical analyses

The statistical analyses (Student test (t-test)) for MTT assay and test with Annexin V and PI were performed with Graph Pad Prism 5.0 (USA). Determination of IC50 test has been used.

P<0.05 were considered significant, while p<0.01 and p<0.001 were considered highly significant.

## 3. Results and Discussion

### 3.1 Structural analysis of nanoparticles

**Fig 1:**
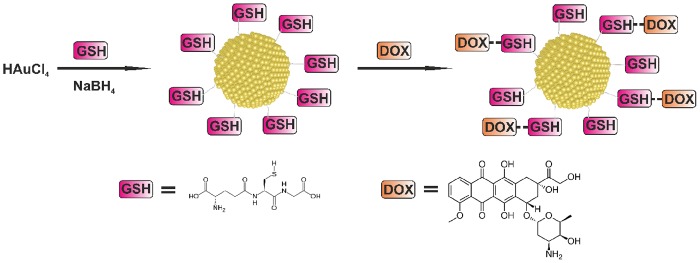
Synthesis scheme for the preparation of Au-GSH and Au-GSH-Dox. In the first stage gold nanoparticles 4.3 +/- 1.1 nm in diameter covered with glutathione molecules are obtained. Then, Doxorubicin conjugation reaction is performed.

**Fig 2:**
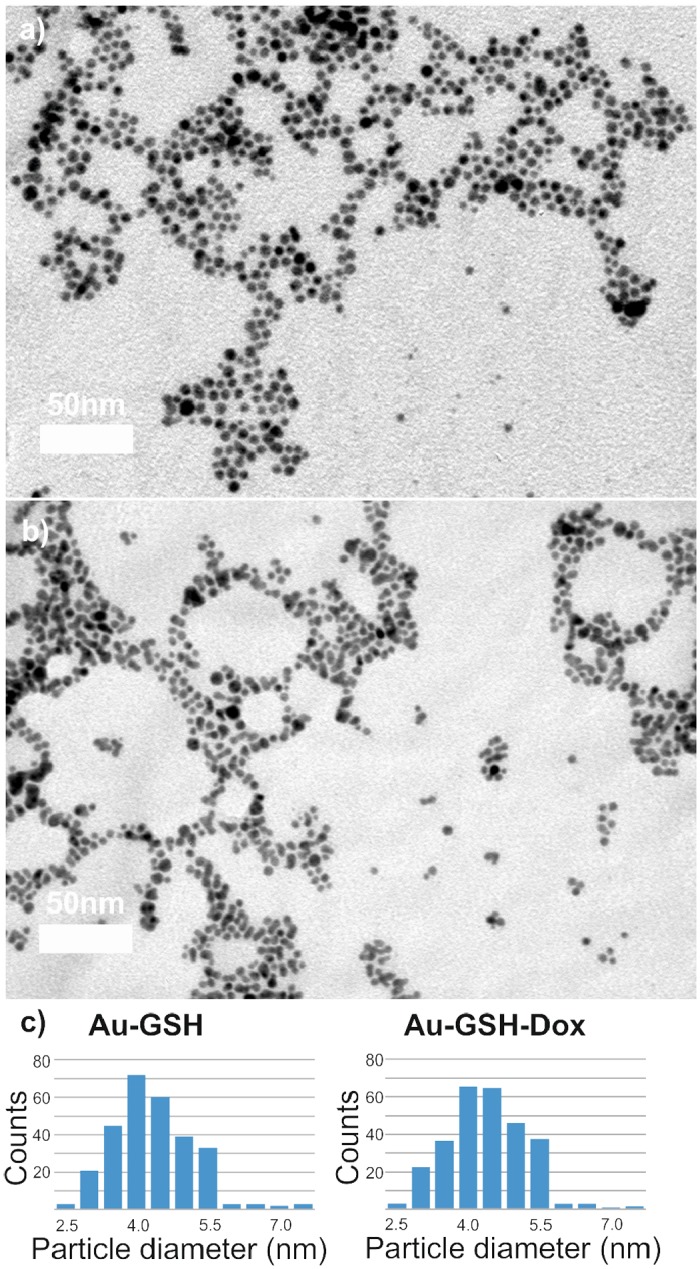
Transmission electron microscopy (TEM) analysis of nanoparticles. Representative TEM micrographs of (a) Au-GSH and (b) Au-GSH-Dox samples. Scale bar is 50 mn. (c) histograms of nanoparticles’ diameter distribution prepared based on TEM micrographs.

**Fig 3:**
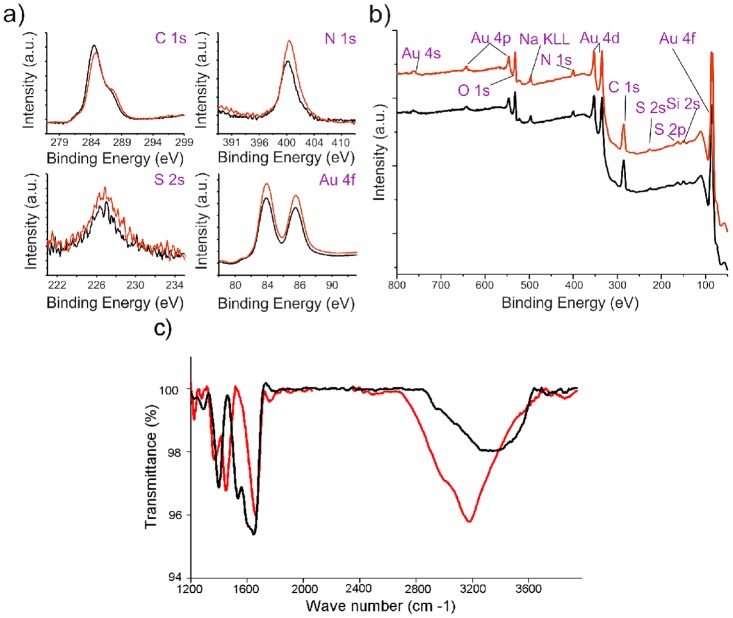
X-ray photoelectron and IR analysis of nanoparticles (Au-GSH—red lines, Au-GHS-Dox—black lines). (a) XPS high-resolution spectra of regions corresponding to Au 4f, C 1s, S 2p and S 2s signals and (b) survey analysis with elements assignment; (c) IR spectra of nanoparticles.

**Fig 4:**
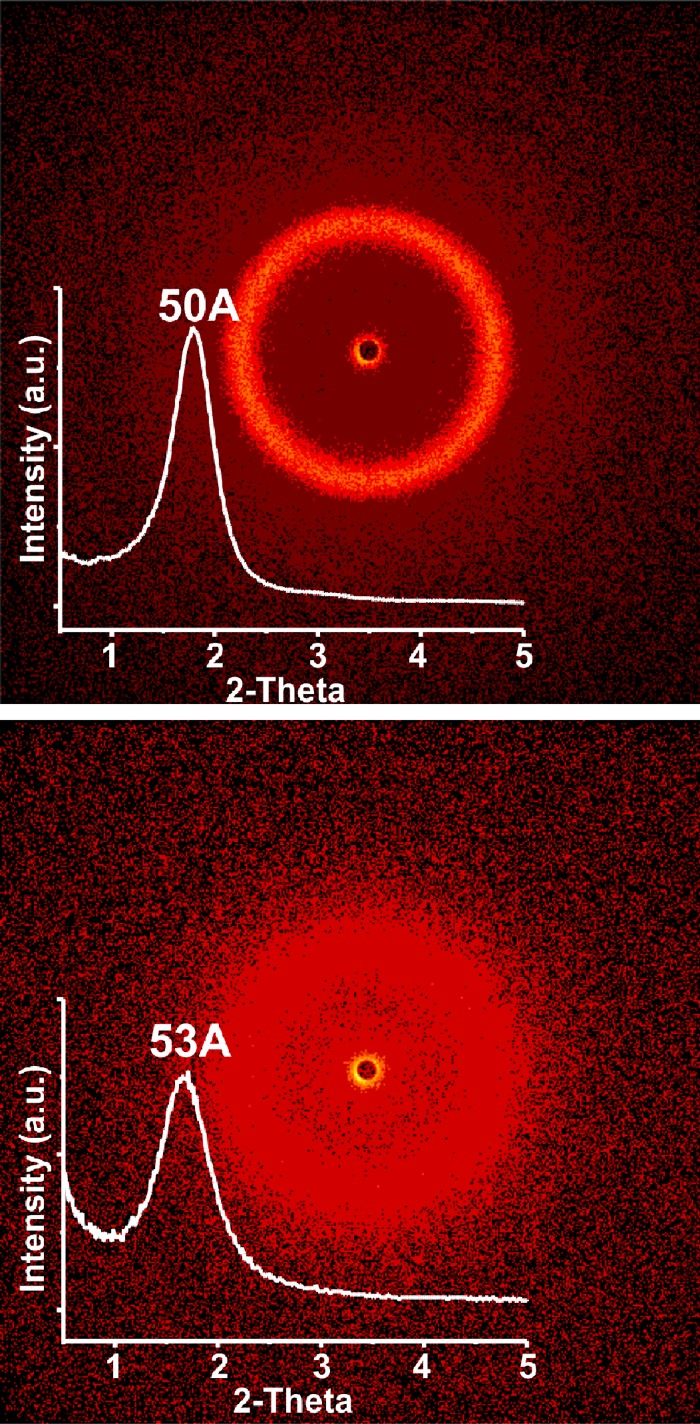
SAXRD analysis. SAXRD analysis of Au-GSH (upper image) and Au-GSH-Dox (lower image) nanoparticles. Insets show 1D scan of the diffractograms.

Synthesis scheme of Au-GSH-Dox nanoparticles is given in Fig 1. To evaluate nanoparticle metallic core shape and size we used transmission electron microscopy (TEM) and small angle X-ray scattering (SAXS). TEM confirmed spherical shape of nanoclusters (Fig 2a and 2b) and allowed to estimate the diameter. Further investigation was performed using SAXS analysis which allowed us to calculate the nanoparticle diameter and confirming TEM results. Importantly, both before (Au-GSH, 4.3 +/- 1.1 nm) and after (Au-GSH-Dox, 4.3 +/- 1.2 nm) modification with doxorubicin nanoparticles exhibit the same mean size. This result confirms that non-covalent conjugation of the drug to the nanoparticle does not influence the chemical nature of nanoclusters, their stability as well as structure. To get further insight into the Au nanoparticles structure, chemistry and composition we used X-ray photoelectron spectroscopy (XPS) measurements. Survey analysis (Fig 3b) revealed the presence of all expected elements in the samples (Au, S, C, N, O, as well as Si coming from the substrate). This confirms successful synthesis of nanostructures and evidences the lack of trace contaminations (no additional signals were detected), that could affect biological experiments. To calculate the number of ligands on nanoparticles surface high-resolution spectra of Au 4f, C 1s, S 2p and N 1s regions were collected. Fig 3a shows the comparison of HR XPS spectra for Au-GSH (red lines) and Au-GSH-Dox (black lines) for the most relevant atoms building these systems. The HR-XPS analysis evidenced that there is difference between Au-GSH and Au-GSH-Dox in quantity of each of the elements. Since XPS technique roughly penetrates few nm deep, we assume that the obtained spectra are representative to the material. Relative areas and positions of the peaks were used to calculate the elemental composition of the obtained materials. XPS proved that ca. 83% of Dox used for Au-GSH modification was bound to the material. To understand the efficient loading of the drug to the surface of nanoparticles it should be considered that GSH has three relevant pKa points: 2.12 (COOH) and 3.53 (COOH) and 8.66 (NH_2_). For Au-GSH conjugation with Dox pH ca. 7 was used, similarly to conditions in which biological tests were performed. In this pH glutathione ligands on nanoparticle surface are predominantly in an ionized form. From the structural and electrochemical point of view such structures can be regarded as similar to the well-studied charged polymer nanoparticles which effectively bind Dox. [44,45] Furthermore, FT-IR was used to confirm successful attachment of Dox to glutathione-stabilized gold nanoparticles (Fig 3c). FT-IR spectra of Au-GSH shows characteristic GSH absorption broad bands around 1660 cm-1 coming from NH bending and C = O stretching modes, while no signal at 1570 cm−1 (C = S) is observed confirming the absence of free GSH in the sample.[46,47] FT-IR analysis of Au-GSH-Dox revealed additional bands, e.g. signals at 1280 (C-O-C stretching), 1404 (C-C stretching) and 1612 cm-1 (NH bending) due to Dox conjugation.[48] Finally, small-angle XRD was used to probe the size of nanoparticles (metallic core together with organic coating, Fig 4). The measured sizes (or in other words interparticle centre-to-centre distance) were 50 and 53Å for Au-GSH and Au-GSH-Dox, respectively. Since Dox conjugation reaction did not affect the metallic core size the change of nanoparticle (as shown by TEM imaging) the observed difference can only be attributed to enlargement of the organic coating of nanoparticles. This further evidences attachment of Dox molecules to Au-GSH.

### 3.2 Cytotoxicity assays

**Fig 5:**
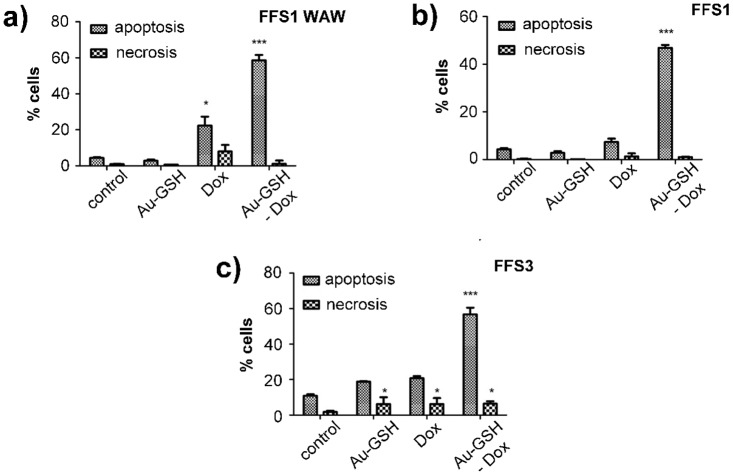
Correlation between cell viability (as measured with MTT assay) and chemotherapeutic dose. Analysis for (a) FFS1WAW, (b) FFS1, (c) FFS3 and (d) FFS5 cell lines. Red and black lines represent Dox and Au-GSH-Dox doses, respectively.

**Fig 6:**
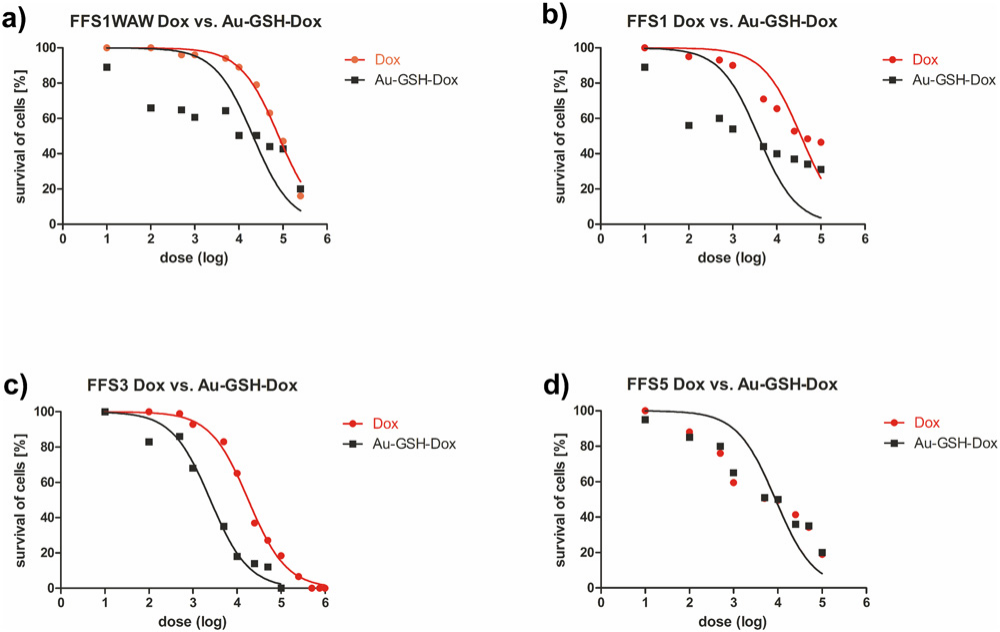
The effect of tested substances (Au-GSH, Dox, Au-GSH-Dox) on apoptosis and necrosis of each cell line: (a) FFS1WAW, (b) FFS1, (c) FFS3. Tested substances were given at concentrations based on the MTT assay results. Statistical analysis was performed using Prism version 5.00 software (GraphPad Software, USA). Unpaired t-test was used, p<0,05 was described as *, p<0.01 was marked as **, p<0.001 was marked as ***

In order to assess the cytotoxic effects of new drugs various *in vitro* tests are used. Depending on the kind of tests: number of necrotic/viable cells, ability for cell proliferation (proliferation index), intact of cell membrane, dyes incorporation into lysosomes, total amount of protein or DNA are analysed using colorimetric, fluorometric, bioluminescence and isotopic methods. [49] One of the most popular methods is MTT assay, which enables measuring half-maximal inhibitory concentration of the tested substances and then allows assessing correlation between inhibition of cells growth and concentration of the tested substances.[50] In our case the calculated IC50 Au-GSH-Dox is 12.5-times, 7.5-times and 2-times lower than IC50 of free Dox for FFS1, FFS3 and FFS1WAW cell lines, respectively (Table 1). However, for FFS5 the relationship is inversed—cytotoxicity of Au-GSH-Dox is slightly higher than of free Dox (Table 1). The results indicate various response for doxorubicin treatment of each cell line (Fig 5). The FFS5 cell line is the most sensitive for doxorubicin, while FFS1 and FFS3 show the highest level of resistance (Table 1). Moreover, the results of MTT assay show that colloid gold nanoparticles in doses from 0.02 μg/ml to 0.5 μg/ml are non-toxic (cell viability >80%, unpublished data). The doses higher than 0.5 μg/ml should not be considered diagnostic as the precipitation of colloid gold nanoparticles from the solution was visible as bluish precipitate. The results show highly significant (p<0.001) increase of the number of apoptotic cells in each of tested cell lines after treatment with Au-GSH-Dox in half maximal inhibitory concentration (IC50) (Fig 6) (S1 Fig). On the contrary, there was no significant increase either of apoptotic or necrotic cells number in FFS1WAW and FFS1 cell lines after treatment with colloid gold nanoparticles (in the same dose as in Au-GSH-Dox) and only a small increase of apoptotic cells in FFS3 cell line (Fig 6) (S1 Fig). These results indicate that higher cytotoxic effect of biocomplex Au-GSH-Dox is not due to cytotoxic effect of Au-GSH alone. These results are in agreement with most of the available data concerning toxicity of colloid gold nanoparticles suggesting that they are safe and non-toxic.[51,52,53] Connor et al.[54] for example reported the lack of toxic effect of Au NPs on human leukaemia cell lines after continuous 3 day exposition. Minati et al.[55] examined physicochemical properties of colloid gold nanoparticles and also indicated that they Au NPs are nontoxic in doses <0.1 mg/ml. Some researchers suggest that toxicity of gold nanoparticles depend not on their concentration but on their size. Pan et al.[19] showed that those of 1–2 nm in diameter are toxic and induce apoptosis in all tested cell lines. Similar results were presented by Goodman et al.[56] who confirmed that nanoparticles with 2 nm in diameter are toxic. Also, the effect of nanoparticle surface ligands should be considered. [18] Our results confirm the thesis that nanoparticles which are bigger than 2 nm in diameter and stabilized with a biogenic molecule are non-toxic. It should be also noted that there is no significant increase of the number of apoptotic cells in FFS1, FFS3 and FFS5 cell lines after treatment with Dox (in the same doses as Au-GSH-Dox). Only for the FFS1WAW cell line slight increase of the number of apoptotic cells in comparison to control group (p<0.05) was observed (Fig 6). Our studies showed that cytotoxic effect of Dox after 24 hour incubation on feline fibrosarcoma cell lines is between 8.67 μg/ml (FFS5) to 69.657 μg/ml (FFS1WAW). There is just one report showing cytotoxic effect on feline fibrosarcoma cell lines for FFA and FFB cell lines isolated in the USA.[57] In the presented studies IC50 Dox for FFA and FFB cell line were 1.4 μg/ml and 1.6 μg/ml respectively. According to these results Williams et al.[57] described their cell lines as sensitive for doxorubicin treatment. However, in our studies IC50 Dox were significantly higher 8.67 μg/ml (for FFS5 cell line), 18.09 μg/ml (for FFS3), 35.389 μg/ml (for FFS1) and 69.657 μg/ml (for FFS1WAW). The obtained results indicate various response of different fibrosarcoma cell lines for doxorubicin. It correlates with the practical knowledge that response for doxorubicin treatment in patients with ISS is debatable and various.[58] Randomised studies on 35 cats with ISS showed that only 38% had positive reaction for doxorubicin treatment (5 cats had total remission and 8 cats had partial remission of the disease). However, many scientists indicate that doxorubicin is totally ineffective and has many adverse side effects.[29,59]

### 3.3 Test with rhodamine 123 and verapamil

There were no differences between cell line (FFS1WAW, FFS1, FFS3, FFS5) in MFI of rhodamine 123 after inhibiting with 10 μM either 20 μM verapamil, which indicates that 10 μM of verapamil is enough to block efflux pumps action. The presented results indicate that the highest efflux pump activity (mainly due to P-gp presence) was observed for FFS1 and FFS3 cell lines. On the other hand, no efflux pump activity was shown for FFS5 cell line. These results correlate with the measured cytotoxicity of studied chemotherapeutics. Cell lines with the highest efflux pumps activity (384 and 541 respectively, Table 1) exhibit the largest difference in Au-GSH-Dox and free Dox action (the cytotoxic effect is 12.5-times and 7-times higher than free Dox for FFS1 and FFS3 cell lines, respectively). For FFS1WAW cell line with medium efflux pumps activity IC50 Au-GSH-Dox was 2-times higher than IC50 Dox. Finally, FFS5 cell line showing no activity of efflux pumps do not respond better to Au-GSH-Dox (Table 1). Our studies show that higher cytotoxic activity of Au-GSH-Dox was observed for feline fibrosarcoma cell lines with P-gp activity. This may indicate that nanoparticles enter cells by endocytosis, avoiding P-gp related MDR as it was suggested by Gu et al.[7] Gu et al. shown that Au-PEG-SS-Dox has higher cytotoxic effect on human liver cancer cell line HepG2R expressing high MDR than free Dox. Similar results presented Minati et al.[55] for human epithelial lung cancer cell line—A549—biocomplex of Dox conjugated to Au NPs had higher cytotoxic effect than the free Dox and higher transport of Au into cell nuclei.

## Conclusions

In this study we have presented a new drug delivery system based on glutathione stabilized, 4 nm diameter gold nanoparticles. Its structure was chosen so as to assure non-toxicity of the material which was then confirmed experimentally. We used the constructed DDS to deliver Dox to feline injection-site sarcoma cell lines. We have demonstrated significantly higher (p<0.01) cytotoxic activity of Au-GSH-Dox than free Dox for 3 (FFS1WAW, FFS1, FFS3) out of 4 feline fibrosarcoma cell lines. The effect has been then correlated to the activity of P-gp system responsible for multi-drug resistance of cancer cells, suggesting that GSH coated Au NPs are good doxorubicin nanocarriers for feline fibrosarcoma cell lines with high P-gp activity. Moreover, due to non-covalent attachment of the chemotherapeutic we can assume that the synthesized system is suitable to a wealth of different drug molecules.

## Supporting Information

S1 Fig Representative cytograms of FFS1WAW, FFS1, FFS3 cell lines double stained with AnnexinV-FITC and PI. Cells were untreated (a) or treated for 4 hours with tested substances: (b) Au-GSH(FFS1WAW 601.2 μg/ml; FFS1 56 μg/ml; FFS3 52 μg/ml), (c) Dox (FFS1WAW 30.6 μg/ml; FFS1 2.8 μg/ml; FFS3 2.6 μg/ml), (d) Au-GSH-Dox (FFS1WAW 30.6 μg/ml; FFS1 2.8 μg/ml; FFS3 2.6 μg/ml). Normal, early apoptotic, late apoptotic and necrotic cells are shown in the cytograms.
(TIF)
