# Correction: Enhancing Anti-Tumor Efficacy of Doxorubicin by Non-Covalent Conjugation to Gold Nanoparticles - *In Vitro* Studies on Feline Fibrosarcoma Cell Lines

**DOI:** 10.1371/journal.pone.0129639

**Published:** 2015-06-01

**Authors:** Michał Wójcik, Wiktor Lewandowski, Magdalena Król, Karol Pawłowski, Józef Mieczkowski, Roman Lechowski, Katarzyna Zabielska

The images for Figs 5 and 6 are incorrectly switched. The image that appears as Fig 5 should be Fig 6, and the image that appears as Fig 6 should be Fig 5. The figure captions appear in the correct order.

**Fig 5:**
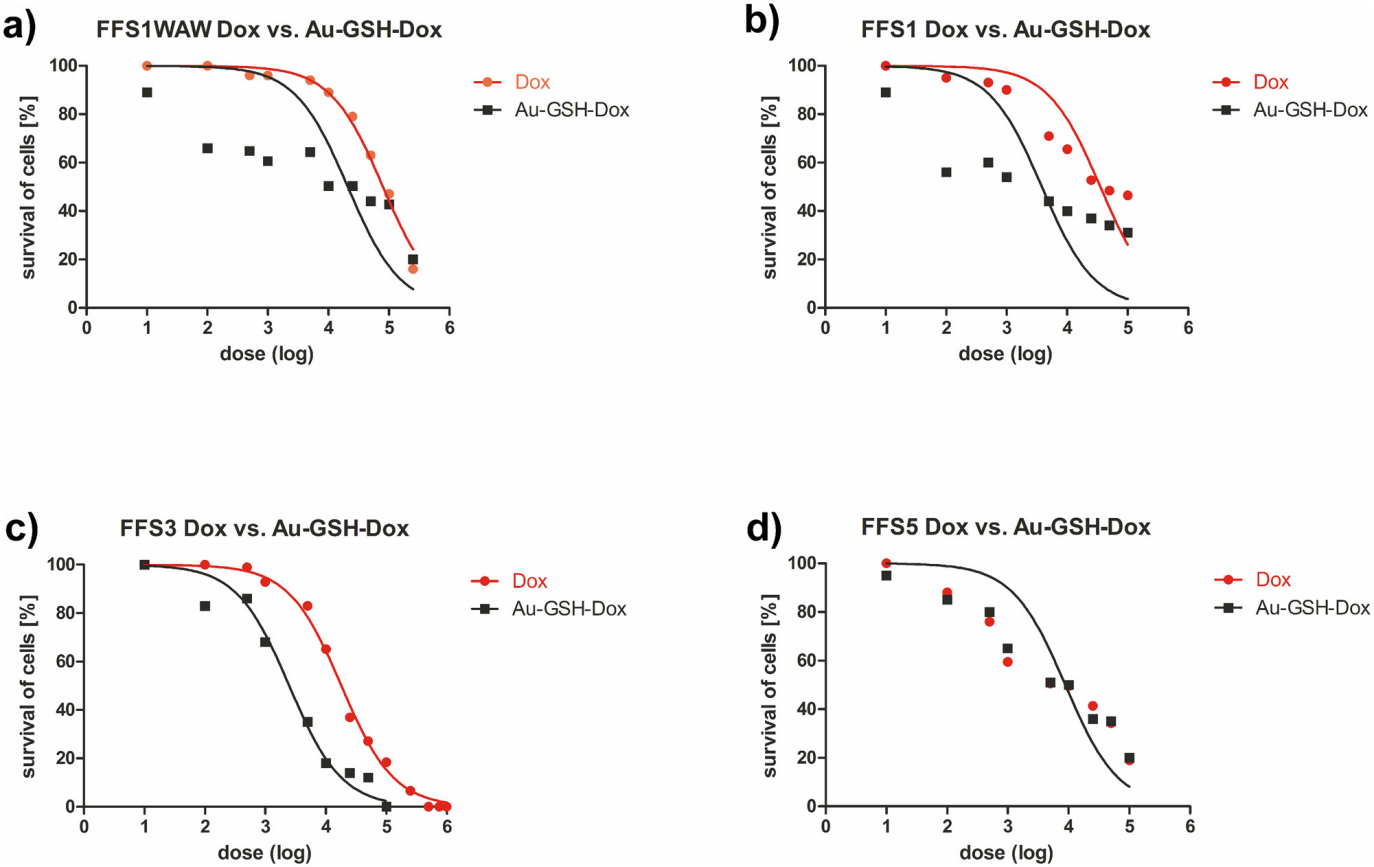
Correlation between cell viability (as measured with MTT assay) and chemotherapeutic dose. Analysis for (a) FFS1WAW, (b) FFS1, (c) FFS3 and (d) FFS5 cell lines. Red and black lines represent Dox and Au-GSH-Dox doses, respectively.

**Fig 6:**
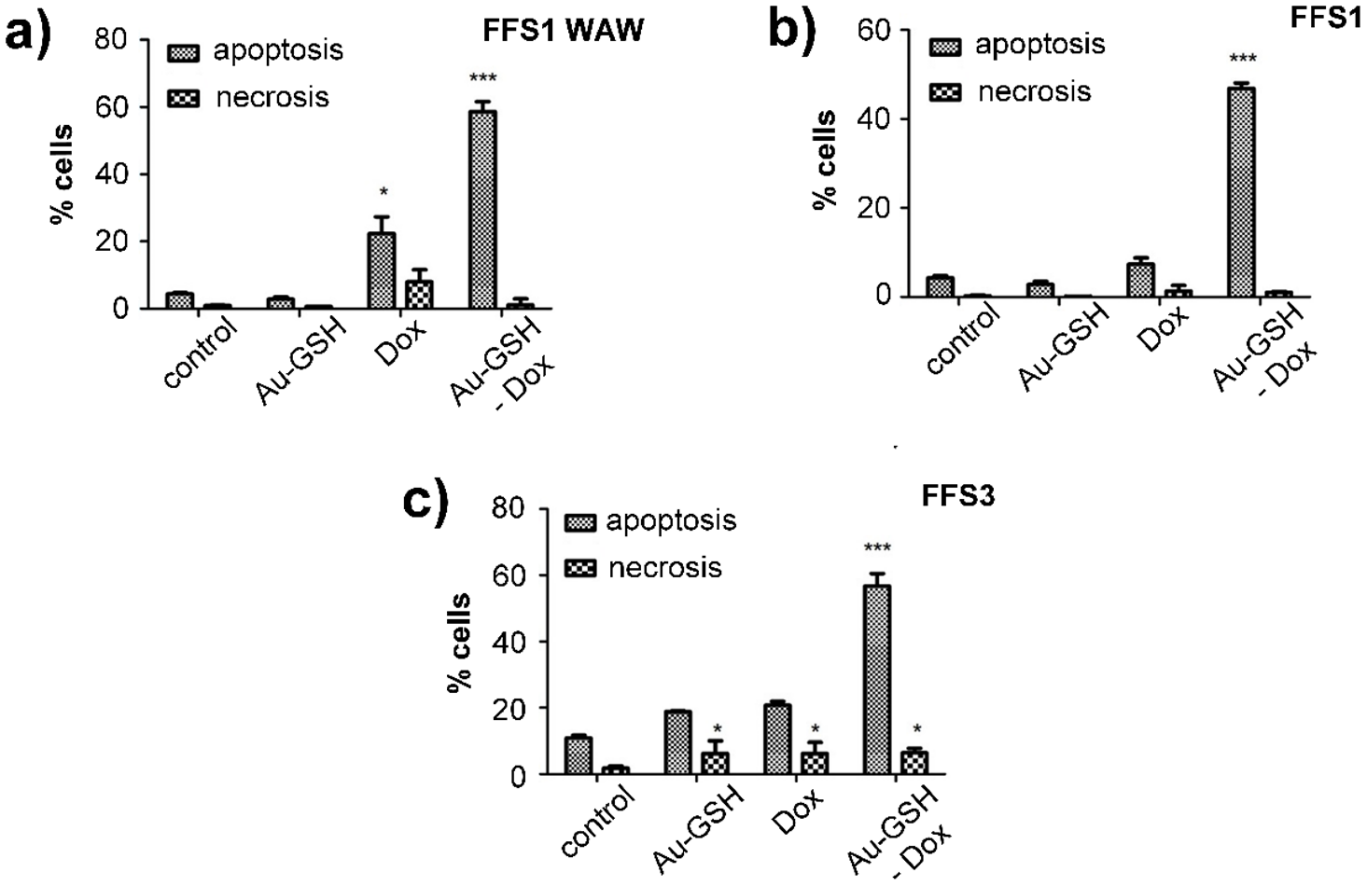
The effect of tested substances (Au-GSH, Dox, Au-GSH-Dox) on apoptosis and necrosis of each cell line: (a) FFS1WAW, (b) FFS1, (c) FFS3. Tested substances were given at concentrations based on the MTT assay results. Statistical analysis was performed using Prism version 5.00 software (GraphPad Software, USA). Unpaired t-test was used, p<0,05 was described as *, p<0.01 was marked as **, p<0.001 was marked as ***
