# Supplementary figures and images for: Enhancing Anti-Tumor Efficacy of Doxorubicin by Non-Covalent Conjugation to Gold Nanoparticles – In Vitro Studies on Feline Fibrosarcoma Cell Lines
Source: PLoS One. 2015 Apr 30;10(4):e0124955. doi: 10.1371/journal.pone.0124955 (PMC4415975; doi:10.1371/journal.pone.0124955)

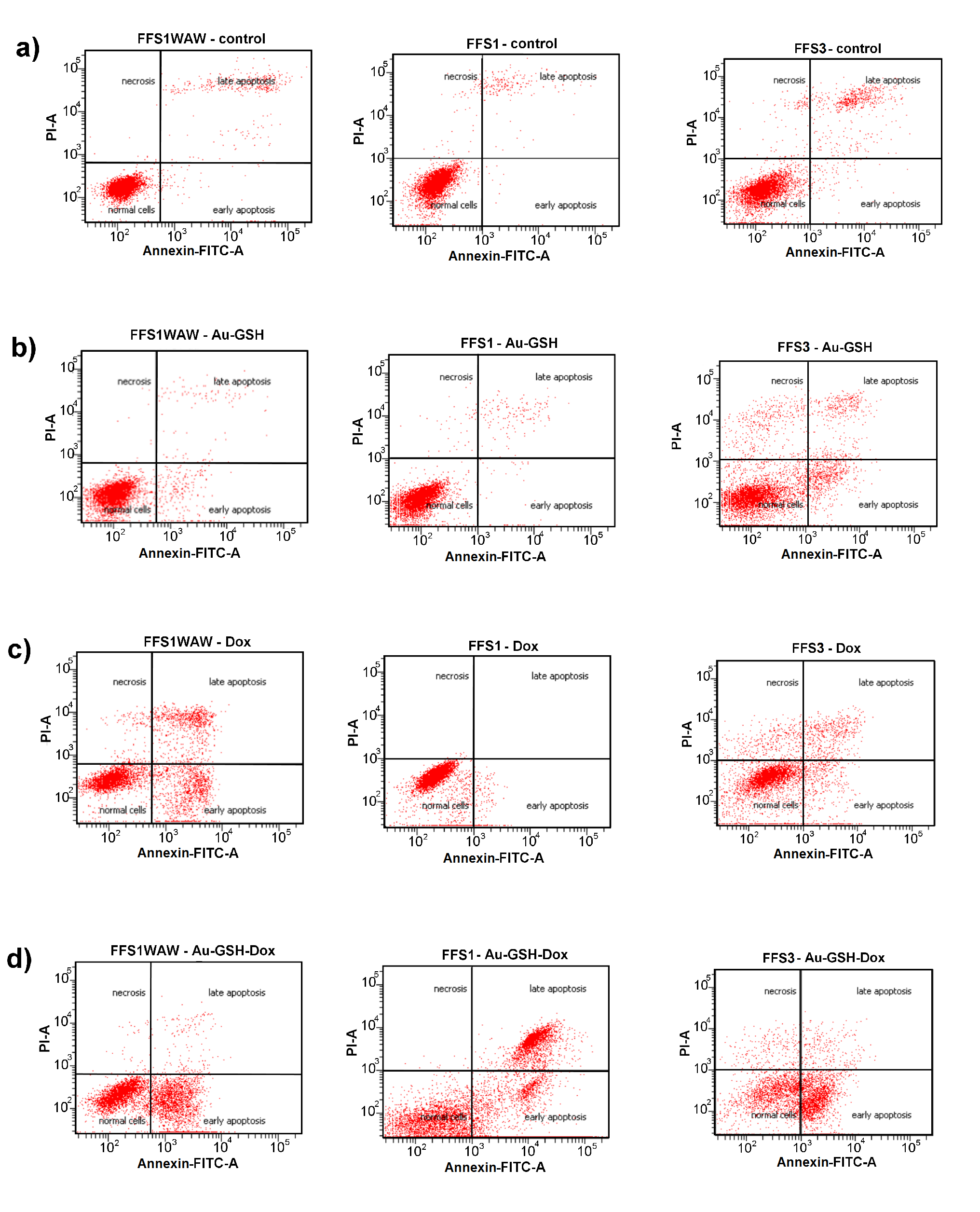

Supplement: S1 Fig — Cells were untreated (a) or treated for 4 hours with tested substances: (b) Au-GSH(FFS1WAW 601.2 μg/ml; FFS1 56 μg/ml; FFS3 52 μg/ml), (c) Dox (FFS1WAW 30.6 μg/ml; FFS1 2.8 μg/ml; FFS3 2.6 μg/ml), (d) Au-GSH-Dox (FFS1WAW 30.6 μg/ml; FFS1 2.8 μg/ml; FFS3 2.6 μg/ml). Normal, early apoptotic, late apoptotic and necrotic cells are shown in the cytograms. (TIF) [file pone.0124955.s001.tif]
